# Standardized 16S Meta‐Analysis Reveals Consistent Host‐Associated Patterns in Laminariales Epiphytic Microbiomes

**DOI:** 10.1002/mbo3.70372

**Published:** 2026-08-17

**Authors:** J. Z. Florez, A. Zárate, P. Bruna, B. Leyton‐Carcaman, V. Molina, M. B. Hengst, C. Camus, M. Abanto, L. Barrientos, A. H. Buschmann

**Affiliations:** ^1^ Centro i~mar, CeBiB and MASH Universidad de Los Lagos Puerto Montt Chile; ^2^ Departamento de Ciencias y Geografía, Facultad de Ciencias Naturales y Exactas and HUB Ambiental UPLA Universidad de Playa Ancha Valparaíso Chile; ^3^ Facultad de Ciencias de la Salud, Instituto de Ciencias Aplicadas Universidad Autónoma de Chile Temuco Chile; ^4^ Núcleo Científico y Tecnológico en Biorecursos (BIOREN) Universidad de La Frontera Temuco Chile; ^5^ Centro de Investigación Oceanográfica COPAS COASTAL Universidad de Concepción Concepción Chile; ^6^ Departamento de Ciencias Farmacéuticas, Facultad de Ciencias Universidad Católica del Norte Antofagasta Chile

**Keywords:** 16S rRNA gene, co‐occurrence networks, epibacterial communities, functional inference, host specificity, macroalgal holobiont

## Abstract

Macroalgae and their associated microbes form tightly integrated holobionts, yet cross‐study comparisons of epiphytic microbiomes remain constrained by heterogeneous sampling designs, primer selection, and analytical pipelines. We performed a harmonized meta‐analysis of publicly available 16S rRNA gene datasets to examine diversity, community structure, and inferred functional profiles within a common analytical framework. A structured search identified 16 studies, totaling 763 samples, from macroalgae and adjacent seawater across four oceanic regions. All datasets were reprocessed using a standardized workflow to ensure cross‐study comparability. The compiled dataset was strongly skewed towards brown macroalgae (Phaeophyceae) and the V4 region of the 16S gene, highlighting both the prominence of Laminariales in current research and the need for methodological harmonization. Within this framework, alpha diversity differed among macroalgal orders, with Fucales hosting the richest epiphytic assemblages, Desmarestiales intermediate diversity, and Laminariales the lowest, while seawater exhibited intermediate values. Community composition was structured by host taxonomic identity and sample type. Within Laminariales, thalli supported assemblages distinct from seawater, sharing a limited taxonomic core and displaying reproducible genus‐level signatures. Trait‐based functional annotation indicated enrichment of aerobic chemoheterotrophy and polysaccharide‐associated metabolisms in Laminariales communities, whereas seawater showed greater representation of nitrogen‐ and sulfur‐related processes. These findings demonstrate that cross‐study reprocessing resolves consistent host‐associated patterns and establishes a quantitative framework for future multi‐omics and experimental investigations.

## Graphical abstract

An integrated bioinformatic pipeline combining taxonomic, functional, and network analyses revealed host‐driven structuring of macroalgal microbiomes. Phaeophyceae hosted distinct epiphytic communities enriched in chemoheterotrophy and polysaccharide degradation, highlighting metabolic coupling, keystone taxa, and a standardized framework for comparative multi‐omics holobiont studies.

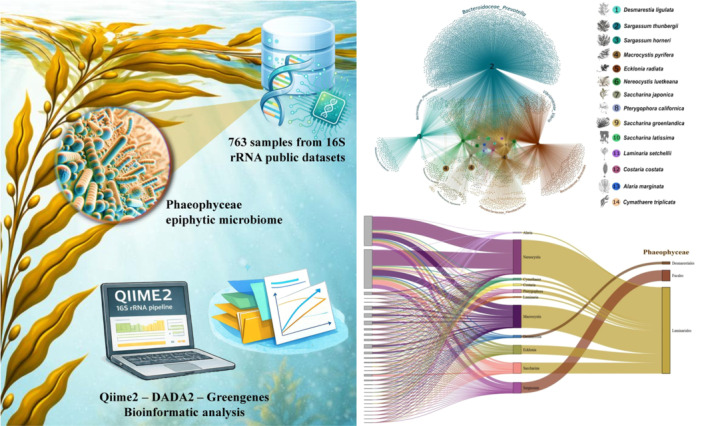

## 1 Introduction

Despite evidence dating back more than 50 years that microorganisms are essential for normal macroalgal growth and development (Pedersén 1968), only in recent decades has a holistic perspective emerged, pointing out that macroorganisms function as ecological units in concert with their associated microbial partners rather than in isolation (Zilber‐Rosenberg and Rosenberg 2008; Egan et al. 2013; Dittami et al. 2021; Saha et al. 2024). These interactions enhance host growth, health, and acclimation to environmental change, with ecosystem‐level consequences for nutrient cycling and habitat structuring (Harley et al. 2012; van der Loos et al. 2019). This integrated entity, the holobiont, has evolved from a conceptual model (Meyer‐Abich 1943) to an empirically grounded framework in evolutionary biology and ecology. While endosymbiosis provides a historical example of intimate host–microbe associations (Margulis 1991; Baedke et al. 2020), the present study adopts an ecological perspective on the macroalgal holobiont, focusing on epiphytic bacteria inhabiting the surfaces of macroalgae habitat‐forming brown seaweeds, particularly kelp‐forming species in the order Laminariales.

Microbial communities associated with macroalgae encompass eukaryotes, archaea, viruses, and bacteria (Wahl et al. 2012), yet bacteria have received the greatest attention and are widely recognized as the principal microbial symbionts of macroalgae (Saha et al. 2024). Epiphytic bacterial assemblages contribute to multiple aspects of macroalgal biology, including the induction of morphogenesis (Wichard 2015; Grueneberg et al. 2016), regulation of spore release and settlement (Morris et al. 2016), defense mechanisms such as antifouling and antibacterial activities (Busetti et al. 2017; Ren et al. 2022), degradation of algal compounds (Menaa et al. 2020) and nutrient acquisition (Singh and Reddy 2014; Nielsen et al. 2019). These functions become particularly critical under environmental stress conditions that challenge the stability and resilience of both host and microbiota (Dittami et al. 2021). Accordingly, the fitness and ecological traits of macroalgal lineages are determined by the complex dynamics and functional significance of their interactions with microbial communities (Paul 1972; Singh and Reddy 2014; van der Loos et al. 2019).

Macroalgae, alongside corals and sponges, are prominent marine holobiont models (Saha et al. 2024). They are photosynthetic, multicellular organisms distributed across diverse ecosystems, providing shelter and food for a wide range of organisms (Graham & Wilcox 1999; Koch et al. 2016). Phylogenetically, they comprise Chlorophyta (green), Rhodophyta (red), and Phaeophyceae within Heterokontophyta (brown) (Guedes et al. 2019). Beyond shared structural polysaccharides (e.g., mannans, xylans, cellulose, hemicelluloses), each group possesses distinctive wall polymers and exudates: alginates/fucoidans in Phaeophyceae, ulvans in Chlorophyta, and agars/carrageenans in Rhodophyta (El‐Manaway and Rashedy 2022), as well as group‐specific biochemical compounds associated with functional traits (i.e., light harvesting, defense, and reproduction) (Øverland et al. 2019; Saha and Weinberger 2019). These biochemical and functional traits likely act as selective filters that shape epiphytic bacterial communities by modulating bacterial attachment, sensing, and growth (El‐Manaway and Rashedy 2022). Accordingly, differences in cell‐wall chemistry and surface infochemicals (Roth‐Schulze et al. 2018) may define distinct holobiont architectures within macroalgae, influencing bacterial recruitment and persistence under variable environmental conditions (Wahl et al. 2012; Guedes et al. 2019; Øverland et al. 2019; Saha and Weinberger 2019). Within these lineages, brown macroalgae (Phaeophyceae), and particularly Laminariales, form extensive forests that dominate temperate rocky reefs across multiple ocean basins. Their large thalli and chemically complex surfaces, rich in alginates and fucoidans, provide a high‐area, chemically heterogeneous habitat for microbial colonization, making Laminariales an ideal model for exploring broad‐scale patterns in epiphytic microbiome assembly, which has, until now, biased the attention they have received.

The prevailing consensus indicates that bacterial community composition varies markedly across host identity, geographic and environmental context, thallus region, and life‐history stage (Lemay et al. 2018; Khan et al. 2024; Syukur et al. 2024). Epiphytic assemblages on macroalgae are typically distinct from those in the surrounding seawater, sharing only a subset of bacterial taxa across hosts and environments (Nielsen et al. 2019; Miranda et al. 2022). Across red, green, and brown macroalgal lineages, these assemblages are shaped by the combined influences of host physiology, surface chemistry, and environmental gradients (Florez et al. 2017; Nahor et al. 2024; Vigil et al. 2024). However, much of our current understanding stems from isolated, methodologically heterogeneous datasets, which hampers efforts to identify “core” or broadly shared bacterial partners within the macroalgal holobiont and to link community composition to host physiology and function (Florez et al. 2017; Gilbert et al. 2018; Chen et al. 2022).

At present, the 16S rRNA gene remains the marker of choice for profiling microbial communities throughout various environments, and reference databases dedicated to this gene continue to expand rapidly (Santos et al. 2020; Bharti and Grimm 2021; Jo et al. 2024). Nevertheless, inconsistencies in sampling design, DNA extraction methods, targeted 16S regions, and bioinformatic workflows can introduce substantial biases in taxon detection and relative abundance estimates, ultimately compromising reproducibility and cross‐study comparability (Dittami et al. 2021; Valencia et al. 2024). Therefore, standardized workflows, coupled with comparative assessments of commonly targeted 16S regions (e.g., V1–V3, V4, V3–V4) and validation through mock communities, are critical to ensure robust and integrative interpretations of microbiome data (Ma et al. 2020). In parallel, comparative syntheses that apply a common downstream pipeline to existing datasets and integrate taxonomic, functional, and network‐based analyses are essential for identifying candidate keystone taxa and elucidating connectivity and assembly patterns within macroalgal holobionts (Layeghifard et al. 2017; Sun et al. 2023). Here, we perform a meta‐analysis of publicly available 16S rRNA datasets using a unified bioinformatics pipeline to compare the diversity and taxonomic–functional structure of epiphytic bacterial communities associated primarily with brown macroalgae (Phaeophyceae) across multiple oceanic regions. Given the uneven representation of 16S rRNA regions across studies, amplicon choice is treated as background metadata rather than a primary explanatory factor in our analyses. Specifically, we address three questions: 1. What 16S rRNA regions are represented in current datasets for Phaeophyceae, and what limitations does this impose on the scope and interpretation of our global synthesis? 2. How do epiphytic bacterial communities differ in diversity and composition among Laminariales host genera and between kelp surfaces and surrounding seawater across the compiled studies? And 3. Are there recurrent taxa, network connectivity patterns, and functional profiles that are consistently associated with Laminariales across the compiled datasets? Overall, we aimed to use a standardized re‐analysis of existing 16S rRNA datasets to identify common and host‐specific features of Laminariales epiphytic microbiomes.

## 2 Materials and Methods

### 2.1 Structured Data Collection

A systematic bibliographic search was conducted using Web of Science, Google Scholar, and the NCBI database to identify articles containing publicly available 16S rRNA epibacterial sequences associated with macroalgal communities. The search terms used were *(macroalgae OR seaweed) AND (bacterial communities OR microbiome OR microbiota OR epibacteria) AND (16S rRNA sequencing OR metabarcoding OR amplicon sequencing)*, and the search included all articles published up to December 2023. The following inclusion criteria were applied: 1. Studies conducted in natural populations of macroalgae; 2. Studies that provided freely accessible bacterial DNA sequences in public repositories; 3. Studies that used Illumina sequencing technology, and 4. Studies that provided the necessary metadata for processing the available DNA sequences. For each study, we retrieved information on host taxonomic group (i.e., Chlorophyta, Rhodophyta, Phaeophyceae), order, genus, and species; sampling matrix (i.e., macroalgal surface vs seawater); oceanic region; and targeted 16S rRNA gene region. Phaeophyceae, and particularly the Laminariales order and seawater, were subsequently used as the main subset for comparisons, whereas Chlorophyta and Rhodophyta were excluded from the principal analysis due to the few available samples that met our selection criteria.

### 2.2 Processing and Standardization of Microbiome Sequencing Reads

Microbiome analysis was conducted using QIIME2 (v2022.8) (Bolyen et al. 2019). Raw sequencing reads were recovered for each study using the SRA Toolkit v3.0.1. Subsequently, the sequences were imported into paired‐end and single‐end Phred 33V2 formats, depending on the survey. Specific trimming and truncation parameters were applied to each study; some studies used the FIGARO tool v1.1.2 (Weinstein et al. 2019) to optimize trimming parameters, whereas others underwent manual parameter selection. Denoising and chimera filtering were performed using the DADA2 plugin (Callahan et al. 2016). Rarefaction curves were generated in each study. Taxonomic analysis was performed using the Greengenes2 database v2022.10 (McDonald et al. 2024), which was selected as the most comprehensive and current reference resource available at the time of the analysis. Custom Naive Bayes (NB) classification models (Wang et al. 2007) were developed, tailored to the specific 16S rRNA gene region used in each study. The extraction sequence regions from the Greengenes2 database corresponded to the primer sets used in each study. These curated sequences were then used to train study‐specific NB classifiers. Each customized model was applied to its dataset to generate taxonomic classifications. Finally, the mitochondrial, chloroplast, and *Eukarya* Amplicon Sequence Variants (ASVs) were removed.

To standardize results across studies, a cutoff threshold was established on each study's rarefaction curve to ensure consistency, and it was then used to rarefy the samples. To make different studies comparable, features were normalized using total sum scaling (TSS * 10^6^), based on the total number of counts per sample. For this work, ASVs were consolidated based on taxonomic classification, treating two ASVs as identical across studies if they matched all seven taxonomic levels: kingdom, phylum, class, order, family, genus, and species. The proportions for each study were then summed to facilitate comparison, unaffected by sequencing depth or sample size differences.

### 2.3 Data Analysis

All analyses were conducted in R (v4.1.1; https://www.r‐project.org/) using the phyloseq package (v1.48.0) following taxonomic consolidation and normalization. Sample distributions across sample types, macroalgal groups, host genera, oceanic regions, and 16S rRNA regions were summarized descriptively. For diversity analyses, ASVs with total counts < 10 and variance < 10% were excluded. Alpha diversity was assessed using the Chao1, Shannon, and inverse Simpson indices to capture complementary aspects of community diversity. Differences among groups were evaluated using Kruskal–Wallis tests with post hoc multiple comparisons. Beta diversity was assessed using PERMANOVA based on Bray–Curtis dissimilarities (vegan package v2.7‐3; 999 permutations), and community patterns were visualized by Principal Coordinate Analysis (PCoA).

Within Laminariales, variance partitioning (varpart, vegan) quantified the contributions of host genus, oceanic region, and their shared component to community variation. To reduce imbalance, only genera present in at least two regions and regions containing at least two genera were retained. Adjusted R^2^ values were calculated, and the significance of pure fractions was tested by partial redundancy analyses (999 permutations).

ASV presence/absence matrices were used to identify shared and unique taxa among host genera and regions. Taxonomic composition at family and genus levels was evaluated using relative abundances, and differentially associated taxa were identified with LEfSe (LDA ≥ 2.0) and interpreted as candidate biomarkers.

To infer putative functional profiles from the taxonomic data, bacterial ASVs were collapsed to the genus level and annotated using the FAPROTAX v1.2.12 database and scripts (www.zoology.ubc.ca/louca/FAPROTAX) (Louca *et al*. 2016), mapping taxa to curated metabolic and ecological categories. FAPROTAX profiles were expressed as relative contributions of each functional group per sample and subsequently summarized by host genus and sample type (Laminariales vs. seawater) to compare dominant functional capacities between macroalgal and planktonic communities.

Based on Spearman rank correlations, network analysis was used to evaluate complexity from co‐occurrence patterns among macroalgal groups. Interactions with correlation coefficients greater than 0.75 and a false discovery rate (FDR)‐adjusted p‐value less than 0.01 were retained for network analysis, enabling the deciphering of microbial taxonomic structure through several property measurements, such as the identification of hubs and key taxa within the community (Barberán et al. 2012). Network topology was characterized using standard metrics, including node and edge counts, modularity, average connectivity, average path length, diameter, betweenness centrality, degree, and closeness centrality. Nodes represented ASVs, and edges corresponded to significant correlations between ASVs. Modules were defined as groups of nodes more densely connected within than between groups. Taxa occupying central topological positions (e.g., module hubs or connectors) were considered candidate keystone lineages (Berry and Widder 2014; Röttjers and Faust 2018). Network visualization and topological analyses were performed in Gephi v0.9.5 (Bastian et al. 2009); https://gephi.org) using the Reingold layout algorithm with 10,000 permutations.

## 3 Results

### 3.1 Global Dataset Overview and Representation of 16S rRNA Regions in Phaeophyceae

**Figure 1:**
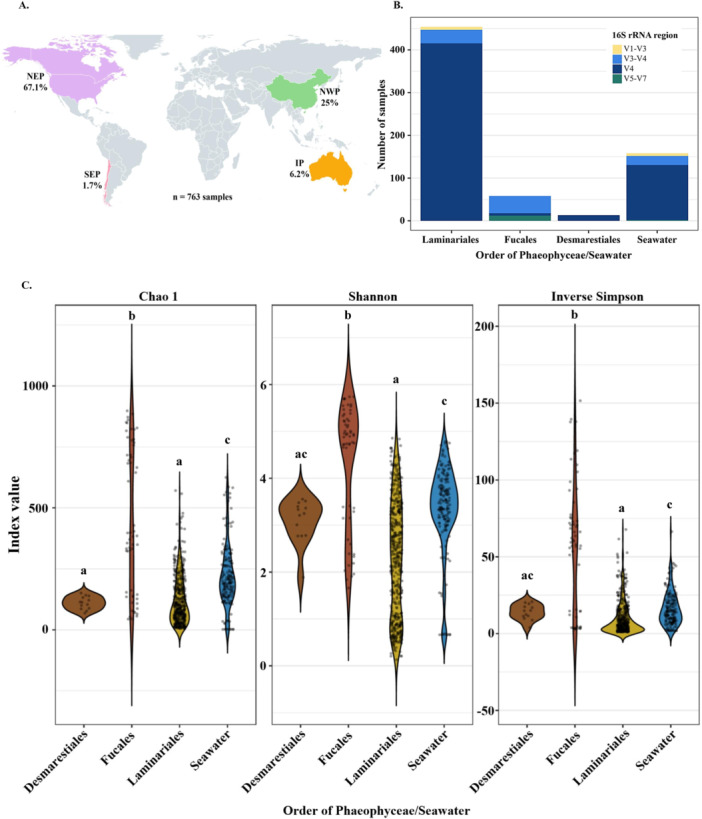
Sampling distribution, marker regions, and alpha diversity of epiphytic bacterial communities. (A) Geographic distribution of the 763 samples included in the meta‐analysis across four oceanic regions: Northeast Pacific (NEP), Southeast Pacific (SEP), Northwest Pacific (NWP), and Indo‐Pacific (IP). (B) Number of samples for each order of Phaeophyceae (Desmarestiales, Fucales, Laminariales) and seawater, stacked by 16S rRNA gene region used for sequencing (V1–V3, V3–V4, V4, and V5–V7). (C) Violin plots showing the distribution of the alpha diversity index (i.e., Chao1 richness, Shannon diversity, and inverse Simpson) for epiphytic bacterial communities associated with each macroalgal order and seawater. Letters represent significant pairwise differences among groups based on Kruskal–Wallis tests followed by the post hoc Tukey HSD test (*p* < 0.001).

The systematic literature search yielded 32 scientific articles, of which 16 met all inclusion criteria (Table S1). These 16 articles contributed 763 samples of macroalgae and seawater (Chlorophyta: 4.3%; Phaeophyceae: 68.8%; Rhodophyta: 5.0%; seawater: 21.9%), distributed across five countries and four oceanic regions (NEP: Northeast Pacific, SEP: Southeast Pacific, NWP: Northwest Pacific, and IP: Indo‐Pacific; Figure 1A, Table S2). Within Phaeophyceae, at the order level, Laminariales comprised 454 samples (59.5%), followed by Fucales (58 samples; 7.6%) and Desmarestiales (13 samples; 1.7%). Across the oceanic regions, Laminariales were present in all four regions (NEP: 70%, SEP: 1.3%, NWP: 6.0%, IP: 9.0%; Table S2). Across all samples, 1,428,700 reads and 118,561 ASVs were obtained, and all rarefaction curves reached a plateau (Figure S1). Phaeophyceae were sequenced predominantly with the V4 region of the 16S rRNA gene (434 samples), followed by V3–V4 (72 samples), V5–V7 (12 samples), and V1–V3 (7 samples) (Figure 1B).

### 3.2 Diversity Patterns in Phaeophyceae Microbiomes

Alpha diversity indices differed among macroalgal orders and seawater (Figure 1C). Fucales exhibited significantly higher alpha diversity than all other macroalgal orders across the diversity metrics evaluated (*p* < 0.001), whereas Laminariales showed the lowest diversity estimates. Seawater samples displayed intermediate diversity levels, while Desmarestiales exhibited diversity patterns comparable to seawater for Shannon and InvSimpson indices, but lower richness estimates according to Chao1 (Figure 1C). At the Laminariales host‐genus level, mean Chao1 richness was highest in *Costaria* and *Cymathaere*, while mean Shannon and InvSimpson values were highest in seawater (Figure S3). Variance partitioning showed that, at the Phaeophyceae level, host order and oceanic region together explained 24% of the variance in community composition (order: 8%; region: 16%), and within Laminariales, host genus and oceanic region together explained 34% (host genus: 18%; region: 12%; shared fraction: 4%; Figure S2). Subsequent analyses, therefore, focused on the host genus within Laminariales.

**Figure 2:**
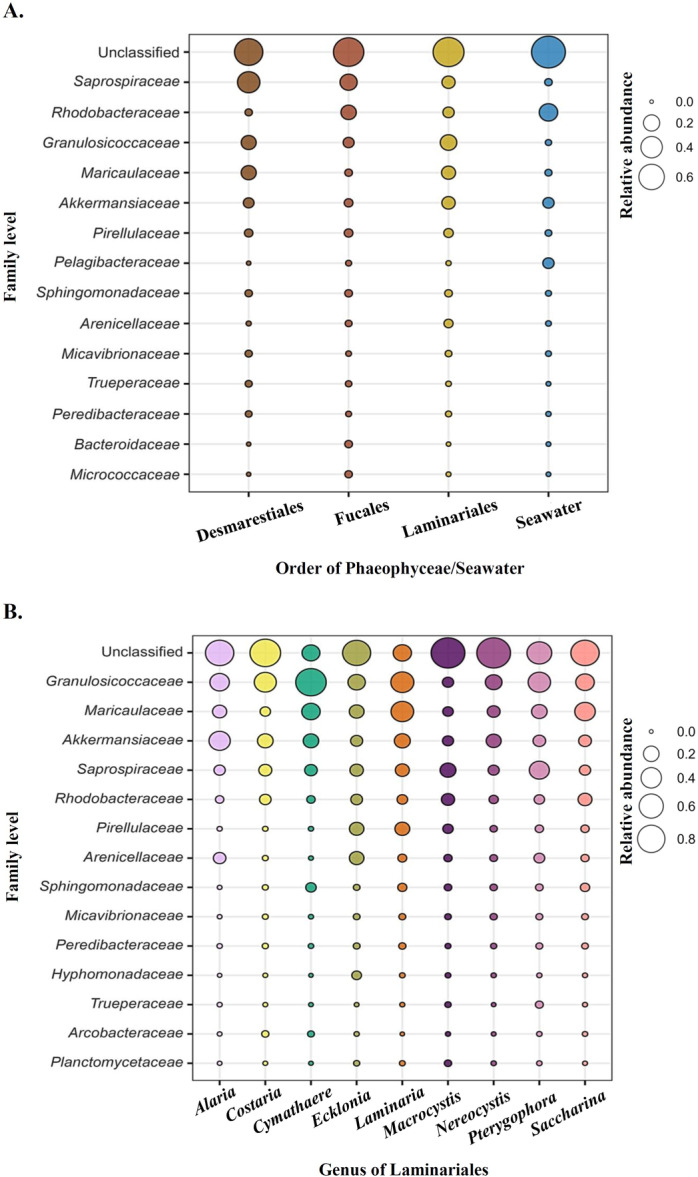
Family‐level composition of epiphytic bacterial communities associated with macroalgae and seawater. (A) Bubble plot showing the relative abundance of the most representative bacterial families across the orders Phaeophyceae (Desmarestiales, Fucales, Laminariales) and seawater. (B) Bubble plot showing the relative abundance of the same bacterial families across Laminariales host genera (i.e., *Alaria, Costaria, Cymathaere, Ecklonia, Laminaria, Macrocystis, Nereocystis, Pterygophora*, and *Saccharina*). Circle size is proportional to the mean relative abundance of each family within each macroalgal order/genus or seawater.

At the family level, Desmarestiales and Laminariales were dominated by Akkermansiaceae (0.03% and 0.06% relative abundance, respectively), Granulosicoccaceae (0.09% and 0.12%), Maricaulaceae (0.09% and 0.07%), and Saprospiraceae (0.26% and 0.06%), whereas seawater samples were characterized mainly by Akkermansiaceae (0.04%), Pelagibacteraceae (0.04%), and Rhodobacteraceae (0.16%) (Figure 2A). Within Laminariales, *Cymathaere* harbored the highest relative abundance of Granulosicoccaceae (0.52%) among host genera, and *Macrocystis* and *Nereocystis* had the highest abundances of unclassified taxa (~0.6% each; Figure 2B).

**Figure 3:**
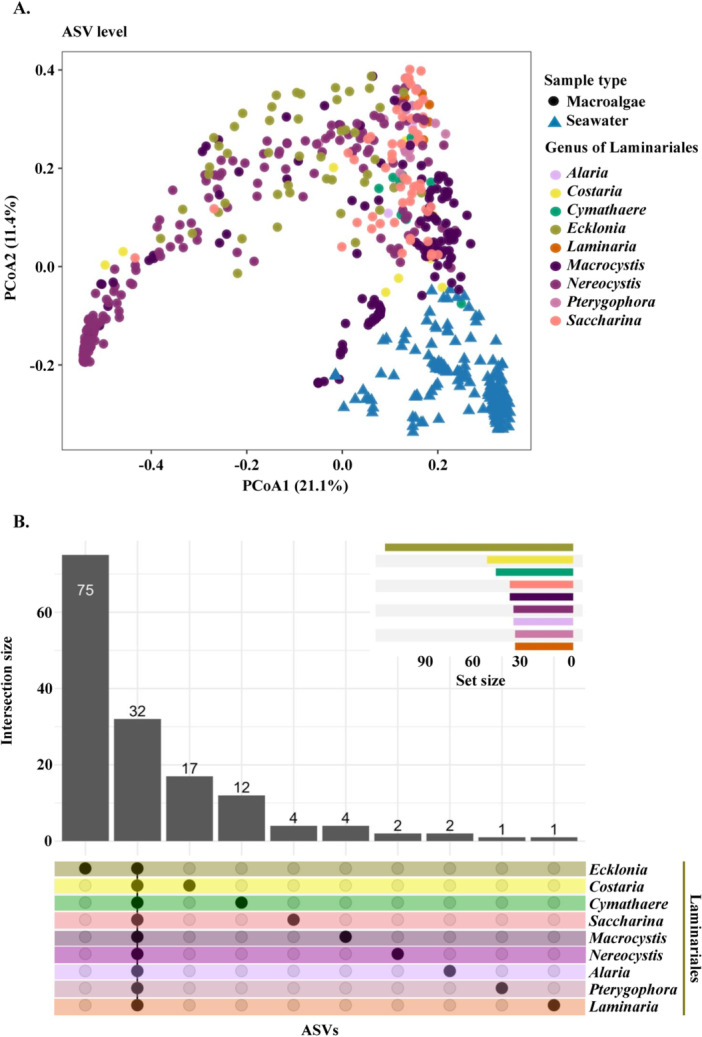
Community structure and shared ASVs among Laminariales host genera and seawater. (A) Principal Coordinates Analysis (PCoA) of epiphytic bacterial communities at the ASV level based on Bray–Curtis dissimilarities. Points represent individual samples, colored by Laminariales host genus and shaped by sample type (circles: macroalgae; triangles: seawater). The first two axes (PCoA1 and PCoA2) explain 21.1% and 11.4% of the variance, respectively. (B) Upset plot showing the distribution of shared and unique ASVs among Laminariales host genera. Horizontal bars (set size) indicate the total number of ASVs detected in each genus, whereas vertical bars (intersection size) indicate the number of ASVs belonging to the specific intersections highlighted in the matrix below. Single filled circles represent genus‐specific ASVs, while connected circles denote ASVs shared among multiple genera.

A PCoA based on ASV‐level Bray–Curtis dissimilarities showed a clear separation between Laminariales epiphytic communities and seawater (PERMANOVA for sample type effect: *F* = 78.88, *R* ^2^ = 0.113, *p* = 0.001), with additional clustering by host genus within Laminariales (Figure 3A; PERMANOVA for host genus effect: *F* = 18.08, *R* ^2^ = 0.195, *p* = 0.001).

### 3.3 Host‐Driven Structuring of *Laminariales* Epiphytic Bacterial Communities

At the ASV level, Laminariales host genera shared a core set of epiphytic taxa while also harboring genus‐specific components (Figure 3B). The largest intersection comprised 32 ASVs detected in all genera (~21% of the total Laminariales ASV richness, *n* = 150 ASVs), with additional intersections of decreasing size representing ASVs shared by subsets of genera. Each host genus also contained exclusive ASVs, and the total number of ASVs differed among genera within the Laminariales dataset (Figure 3B). *Ecklonia* harbored the largest pool of unique ASVs (75 exclusive ASVs; set size > 90 ASVs), followed by *Costaria* (17) and *Cymathaere* (12), whereas the remaining genera had < 4 unique ASVs (set size 30–60 ASVs; Figure 3B).

**Figure 4:**
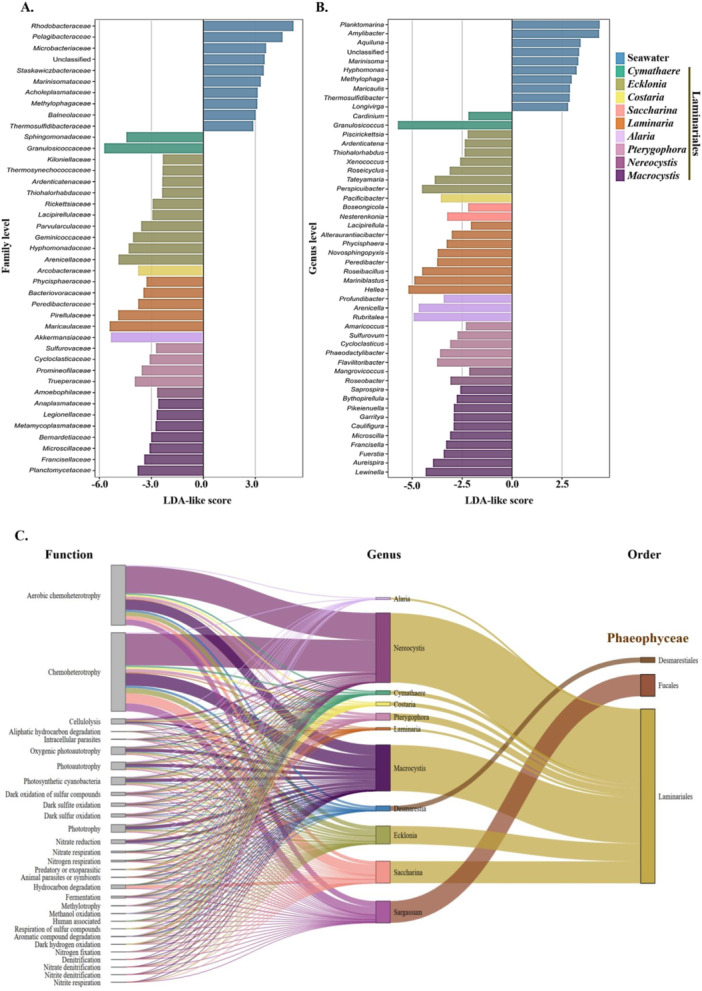
Linear Discriminant Analysis Effect Size (LEfSe) results at the (A) family and (B) genus level comparing epiphytic communities among Laminariales host genera/seawater. Bars represent bacterial families with significant differential abundance, and bar length (LDA‐like score) indicates the magnitude of the association. Colors correspond to the host genus/seawater with which each taxon is associated. (C) Sankey diagram showing functional annotations of epiphytic bacterial communities based on FAPROTAX. The width of each stream is proportional to the relative contribution of ASVs assigned to each functional category within a given host genus and order. Detailed species‐level FAPROTAX functional profiles corresponding to the genera shown here are available in Table S5.

Differential abundance analysis with LEfSe identified families and genera that were preferentially associated with Laminariales host genera or with seawater (Figure 4). At the family level, Laminariales host genera showed between 2 and 10 biomarker families each: *Macrocystis* was enriched in Planctomycetaceae and Francisellaceae (seven biomarkers; LDA > 3.4), *Laminaria* in Maricaulaceae and Pirellulaceae (5; LDA > 4.9), *Ecklonia* in Arenicellaceae and Hyphomonadaceae (10; LDA ≈ 4.3), and *Cymathaere* in Granulosicoccaceae and Sphingomonadaceae (2; LDA ≈ 4.4), whereas seawater had 10 biomarker families dominated by Rhodobacteraceae and Pelagibacteraceae (LDA > 4.5) (Figure 4A). At the genus level, *Macrocystis* was characterized by *Lewinella* and *Aureispira* (10 biomarkers; LDA ≈ 4), *Laminaria* by *Hellea*, *Mariniblastus*, and *Roseibacillus* (8; LDA > 4.5), *Ecklonia* by *Perspicuibacter* (7; LDA ≈ 4.5), *Cymathaere* by *Granulosicoccus* (2; LDA ≈ 5.7), and seawater by *Planktomarina* and *Amylibacter* (10; LDA > 4.3) (Figure 4B).

### 3.4 Core Taxa, Connectivity, and Functional Profiles of Laminariales Microbiomes

FAPROTAX‐based annotations of Phaeophyceae‐associated bacterial assemblages (Figure 4C; Table S5) revealed a limited set of dominant functional groups with contrasting contributions among Laminariales hosts and seawater. Hydrocarbon degradation was mainly associated with *S. japonica*. Chemoheterotrophy and aerobic chemoheterotrophy were the most common functions among Laminariales, occurring in *S. japonica*, *Nereocystis luetkeana*, *M. pyrifera*, and *Ecklonia radiata*. Phototrophy and photoautotrophy were most frequent in *M. pyrifera* and *E. radiata* and were also present in *N. luetkeana*. Nitrogen‐related functions (nitrate reduction, nitrogen respiration) were primarily annotated for *M. pyrifera*, whereas cellulolysis was annotated for both *M. pyrifera* and *N. luetkeana*. The same functional groups were detected in seawater communities, which also showed higher relative contributions of nitrogen‐ and sulfur‐transforming processes and phototrophy, whereas macroalgal samples were dominated by aerobic chemoheterotrophy and chemoheterotrophy (Figure S4).

**Figure 5:**
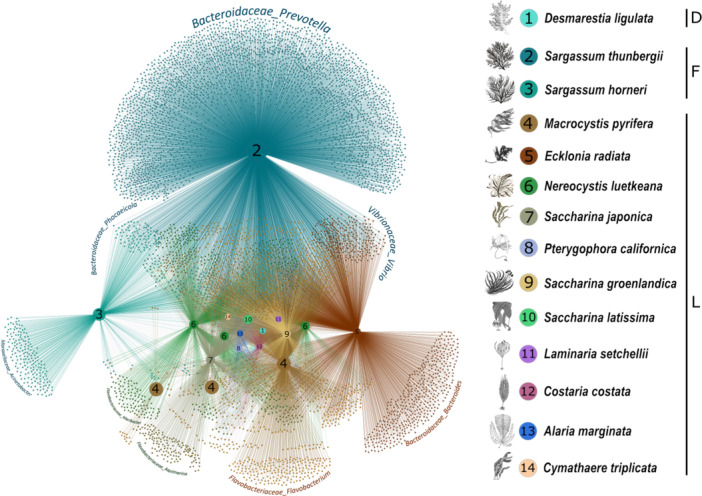
Network based on presence–absence data of Phaeophyceae‐associated ASVs. Nodes correspond to individual macroalgal species. For ease of interpretation, colored nodes with numbers (1–14) represent macroalgal hosts, while small colored nodes correspond to bacterial taxa associated with each host. Edges indicate associations between macroalgae and bacterial families. The size of the host nodes is proportional to their connectivity within the network. Distinct clusters reveal host‐specific bacterial assemblages, whereas overlap among clusters suggests the presence of bacterial taxa shared across multiple macroalgal species. The letters indicate the order of Phaeophyceae to which the genus belongs (D: Desmarestiales; F: Fucales; L: Laminariales).

Presence–absence networks revealed distinct bacterial association patterns between Phaeophyceae hosts and seawater. In the Phaeophyceae network (Figure 5), ASVs formed a compact, highly connected core dominated by Bacteroidaceae, Vibrionaceae, and Flavobacteriaceae. Host‐specific associations were evident, particularly for the *Fucales* species *Sargassum thunbergii* and *Sargassum horneri*, which were mainly linked to Bacteroidaceae, with *Prevotella* and *Phocaeicola* contributing ~46.4% in *S. thunbergii*. Among Laminariales, *Ecklonia radiata* was associated with Bacteroides (~12%), whereas *Macrocystis pyrifera* showed the strongest association with Flavobacteriaceae genera (i.e., *Flavobacterium*, *Aquimarina*, and *Maribacter*; ~16.9%). In *Saccharina japonica*, Flavobacteriaceae accounted for ~5.5% of network associations (Figure 5). In contrast, the seawater network (Figure S5) was characterized by hubs assigned to *Vibrio*, *Luminiphilus*, *Flavobacterium*, the MWS24 clade, and *Lewinella*, forming a more radially distributed topology.

**Figure 6:**
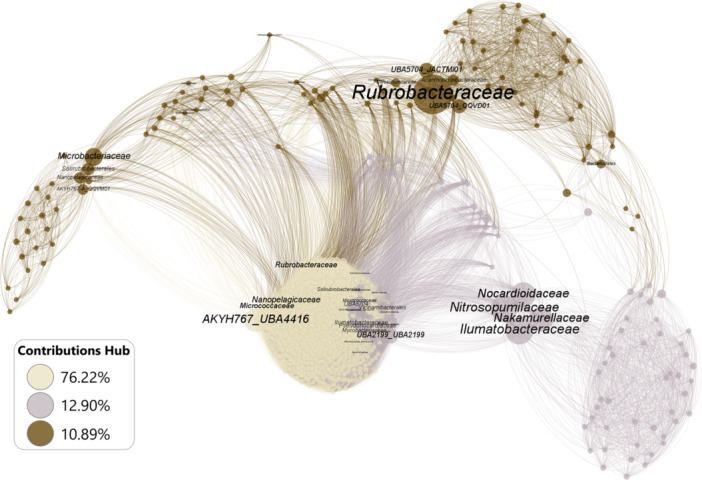
Network of microbial co‐occurrence patterns in bacterial communities at the family level associated with the Phaeophyceae group. The nodes (bubbles) represent the ASVs, and edges (lines) represent the strong correlations (Spearman's *p* > 0.75) and statistical significance (FDR‐adjusted *p* < 0.01). Nodes were colored according to their modularity class, which indicates the module or community to which each node belongs, a property often associated with high clustering coefficients. The size of each node is proportional to the betweenness centrality value (see Table S3).

Finally, co‐occurrence patterns of epiphytic bacterial communities associated with Phaeophyceae and seawater were then evaluated using a correlation matrix. Within the full macroalgal network, Rubrobacteraceae emerged as the principal hub family, forming a large, centrally positioned module that connected numerous ASVs across hosts. Additional highly connected clusters were formed by families such as Microbacteriaceae, Nocardioidaceae, and Nitrosopumilaceae, which also occupied prominent positions in the network (Figure 6; see Table S3). The resulting network between macroalgal groups and seawater revealed a highly interconnected module (light blue) that contained the majority of ASVs and exhibited extensive connectivity among taxa (Figure S6; see Table S4). Within this module, families such as Miltoncostaeaceae, Nitrosopumilaceae, Bacteroidaceae, Muribaculaceae, and members of Actinobacteriota displayed high centrality and large node sizes. Several additional modules were identified and were clearly separated from the main cluster, likely representing bacterial assemblages selectively enriched by specific macroalgal hosts or environmental conditions. Smaller peripheral modules (i.e., brown, olive, and gray) also exhibited strong internal connectivity, suggesting niche‐specific microbial consortia with limited interactions with the broader community. The seawater‐associated community formed a distinct and highly cohesive cluster characterized by numerous closely connected ASVs and minimal connectivity with macroalgae‐associated modules, highlighting a marked differentiation between free‐living and host‐associated bacterial assemblages (Figure S6).

## 4 Discussion

### 4.1 Global Dataset Overview and Representation of 16S rRNA Regions in Phaeophyceae

Although most studies on macroalgae now conceptualize the holobiont as a single ecological and functional unit composed of the host and its microbial partners (Saha et al. 2024) our current understanding of these associations across macroalgal lineages still offers only a partial view of the complexity present in natural systems (Paul 1972; Wahl et al. 2012). Critical knowledge gaps remain regarding which microbial constituents contribute to specific functional traits and how interaction patterns vary among different macroalgal hosts.

Microorganisms, the most diverse form of life on Earth, remain largely unknown because a large fraction remains unculturable (Rinke et al. 2013). This has driven the widespread use of culture‐independent approaches based on marker genes, such as 16S rRNA, which enable microbial diversity, distribution patterns, and ecological functions to be resolved at higher taxonomic and functional levels (Willis et al. 2019). However, the choice of the 16S rRNA region can bias taxon detection and relative abundances, leading to under‐ or over‐representation of lineages (Abellan‐Schneyder et al. 2021). In our meta‐analysis, most Phaeophyceae samples were sequenced using the V4 region, with only a minority targeting V3–V4, V5–V7, or V1–V3, mirroring the dominant use of V4 in marine microbiome surveys (Mizrahi‐Man et al. 2013). Fragments from this region tend to exhibit low error rates because they are flanked on the 3′ side by semi‐conserved segments (Abellan‐Schneyder et al. 2021), which helps explain their versatility in capturing a broad taxonomic range.

Since the empirical distribution of markers is so strongly skewed towards V4, our dataset does not permit a robust quantitative comparison of the performance of different 16S rRNA regions. Instead, the answer to our first question is primarily descriptive: current Phaeophyceae datasets are overwhelmingly V4‐based, with other regions sparsely represented, which constrains formal cross‐region benchmarking. The main contribution here is to document which 16S rRNA regions have been used to characterize Phaeophyceae‐associated communities and to highlight the need to harmonize primer choice and sequencing strategy in future work. All samples were processed through a unified bioinformatic pipeline and rarefied to a common sequencing depth, which reduces artefacts associated with sequencing depth and workflow differences and improves cross‐study comparability (Weiss et al. 2017; Knight et al. 2018), as demonstrated in great, coordinated efforts such as the Earth Microbiome Project (Thompson et al. 2017). Nonetheless, residual heterogeneity in upstream study design (e.g., DNA extraction protocols, primer pairs, sampling, and storage schemes) is unavoidable in retrospective microbiome meta‐analyses, even when a common downstream pipeline is applied, and must be considered when interpreting broad cross‐study patterns (Wang and Lêcao 2020). Together, these patterns emphasize that future comparative work on macroalgal microbiomes would benefit from continued standardization of 16S rRNA regions and analytical pipelines to improve cross‐study comparability (Ma et al. 2020; Dittami et al. 2021; Valencia et al. 2024).

### 4.2 Diversity Patterns in Phaeophyceae Microbiomes

Our compilation confirms that current datasets on epiphytic bacteria are strongly biased towards brown macroalgae, particularly habitat‐forming Phaeophyceae such as *Ecklonia*, *Macrocystis*, and *Saccharina* (Marzinelli et al. 2024). This likely reflects the ecological prominence of these canopy‐forming species as ecosystem engineers, which create structurally complex, high‐area habitats capable of supporting diverse microbial niches (Singh and Reddy 2016). Consequently, Phaeophyceae, and within them Laminariales, dominate the available sequence data and provide the most robust basis for global comparisons. It is important to note that the geographic and taxonomic representation of the dataset was additionally constrained by the availability of publicly accessible sequencing datasets accompanied by sufficient metadata to enable standardized re‐analysis. As a result, some regions and host taxa remain underrepresented despite the existence of published microbiome studies.

Across orders, alpha diversity exhibited a clear gradient: Fucales harbored the richest and most even epiphytic bacterial communities, Laminariales the lowest diversity, and seawater showed intermediate values, with Desmarestiales overlapping seawater in diversity but with reduced richness. The high diversity associated with Fucales is consistent with the view that architecturally complex thalli endowed with chemically rich surface metabolomes can sustain a broad array of microhabitats and organic substrates for colonization (Lachnit et al. 2010; Wahl et al. 2012; Roth‐Schulze et al. 2018). Reviews of the chemical ecology of macroalgae in the order Fucales and of algal polyphenols further highlight the abundance of phlorotannins and other bioactive compounds in this group, which can mediate dense, chemically structured holobionts (Fernando et al. 2022; Rousseau et al. 2024). In contrast, the comparatively lower diversity within Laminariales may indicate stronger host filtering, in which specific surface chemistries and defense traits restrict successful colonization despite the large available surface area (Küpper et al. 2008; Lane and Kubanek 2008). This interpretation is consistent with recent multi‐omics work on *Saccharina latissima*, which shows a tight coupling between the kelp surface metabolome and a relatively specific community of epiphytic bacteria (Adouane et al. 2025).

Within Laminariales, genera such as *Costaria* and *Cymathaere* showed the highest ASV richness, whereas seawater displayed the greatest evenness and diversity in Shannon and inverse Simpson indices. This pattern supports the idea that macroalgal surfaces act as selective filters, enriching lineages from the surrounding planktonic pool, while seawater maintains a broader, more even taxonomic spectrum (Saha et al. 2024). Variance partitioning complements this view by showing that, while both host order and oceanic region contribute to community variation in Phaeophyceae, within Laminariales, host genus alone explains a substantial fraction of the variance and is comparable in magnitude to the effect of region. Taken together, our results and previous work on brown‐algal surface chemistry suggest that differences in thallus architecture, metabolite profiles, and defensive traits among orders and genera contribute to the observed gradient in epiphytic bacterial diversity across Phaeophyceae.

### 4.3 Host‐Driven Structuring of Laminariales Epiphytic Bacterial Communities

Ordination analyses revealed consistent separation between Laminariales epiphytic communities and surrounding seawater, together with clear clustering by host genus. Thus, Laminariales surfaces do not passively mirror the ambient planktonic pool but instead appear to retain specific fractions of the regional bacterial metacommunity (Singh and Reddy 2014; Marzinelli et al. 2015; Roth‐Schulze et al. 2018). Across the Phaeophyceae species examined, we observed host‐specific bacterial combinations, indicating strong host filtering at genus and species levels. The contrasting microbiomes of *S. thunbergii* and *S. horneri*, despite overlapping regional ranges, exemplify this pattern and suggest genus‐level niche partitioning even among closely related hosts. Likewise, *M. pyrifera* supported a distinctive suite of Flavobacteriaceae (i.e., *Flavobacterium*, *Aquimarina*, and *Maribacter*) that did not co‐occur in comparable combinations in other hosts.

The coexistence of a reduced core of epiphytic ASVs shared by all Laminariales genera with a larger set of genus‐exclusive ASVs is consistent with patterns reported in other kelp microbiomes, where a small group of bacterial taxa is consistently retained despite substantial turnover (Lemay et al. 2018). A small‐shared core likely represents lineages able to tolerate or exploit common Laminariales traits, whereas the host‐exclusive fraction reflects the combined influence of host‐specific traits and local environmental contexts (Wahl et al. 2015). This limited taxonomic core may also indicate functional redundancy, whereby distinct bacterial lineages contribute similar ecological functions (Burke et al. 2011; Louca et al. 2018). Consistent with this interpretation, the inferred functional profiles in our study were more conserved than the taxonomic composition across Laminariales hosts, suggesting that key ecological functions may be maintained despite variation in community membership.

Taxonomic patterns further support host‐driven structuring. At the family level, Desmarestiales and Laminariales were dominated by Akkermansiaceae, Granulosicoccaceae, Maricaulaceae, and Saprospiraceae, whereas seawater was characterized by pelagic clades such as Rhodobacteraceae and Pelagibacteraceae, in line with their dominant roles in marine bacterioplankton (Morris et al. 2002; Thompson et al. 2017). Biomarker analyses further showed that each Laminariales genus is associated with distinct diagnostic taxa, for example, Granulosicoccaceae and *Granulosicoccus* in *Cymathaere*, or filamentous Bacteroidota such as *Lewinella* and *Aureispira* in *Macrocystis*. These patterns are consistent with the microbial gardening framework, in which macroalgal hosts structure their microbiomes through surface chemistry, exudates, and species‐specific metabolite environments, including phlorotannins and other bioactive compounds (Rickert et al. 2015; Singh et al. 2017; Minich et al. 2018).

### 4.4 Functional Connectivity and Candidate Keystone Taxa in Laminariales Microbiomes

Despite clear host‐specific taxonomic signatures, Laminariales and seawater share a relatively conserved set of inferred functional capabilities (Louca et al. 2018). FAPROTAX annotations revealed that chemoheterotrophy and aerobic chemoheterotrophy dominated across Laminariales hosts, particularly in *S. japonica*, *N. luetkeana*, *M. pyrifera*, and *E. radiata*, whereas seawater communities showed relatively higher contributions of nitrogen‐ and sulfur‐transforming processes and phototrophy. Individual hosts also exhibited distinct functional emphases: hydrocarbon degradation was mainly associated with *S. japonica*, nitrogen‐related functions (i.e., nitrate reduction, nitrogen respiration) were concentrated in *M. pyrifera*, and cellulolysis was enriched in *M. pyrifera* and *N. luetkeana*. These contrasts suggest that different Laminariales genera recruit partially overlapping bacterial guilds that sustain core heterotrophic functions, while specific hosts additionally support functions linked to nitrogen metabolism and structural polysaccharide turnover.

These functional patterns are consistent with the idea that macroalgal holobionts converge on key metabolic roles, such as organic‐matter remineralisation, polysaccharide degradation, and nutrient cycling, even when their taxonomic composition differs. Previous studies have shown that recurrent bacterial lineages, including representatives of Vibrionaceae, Flavobacteriaceae, and Rhodobacteraceae, often contribute to heterotrophy, antimicrobial responses, and algal polysaccharides degradation, positioning them as functional guilds that support algal health and nutrient dynamics (Campbell et al. 2011; Zozaya‐Valdes et al. 2015). Similarly, our data indicate that Laminariales surfaces are consistently enriched in heterotrophic functions relative to seawater, whereas planktonic communities retain a broader spectrum of nitrogen‐ and sulfur‐related processes. Together, these patterns suggest that Laminariales‐associated bacteria form functionally structured subsets of the surrounding microbial pool, tuned to exploit algal exudates and surface‐derived substrates, while seawater communities maintain a more generalized functional spectrum. This agrees with metagenomic evidence showing that functional gene repertoires, rather than species identity alone, may underpin the stability of macroalgal microbiomes across large spatial scales (Tully et al. 2018).

Building on this functional perspective, our study assumes that candidate keystone microbes can be identified by applying interaction‐network approaches (Paix et al. 2020) to existing 16S rRNA metabarcoding datasets (Florez et al. 2019; Wood et al. 2022). The co‐occurrence network constructed for Phaeophyceae and seawater revealed a highly connected architecture with low modularity and several dense modules, and showed a clear separation between macroalgal and seawater nodes, reinforcing the idea that macroalgal surfaces host distinct, internally cohesive bacterial assemblages relative to the surrounding water column (Florez et al. 2017, 2019; van der Loos et al. 2019; Saha et al. 2024). Within this network, Rubrobacteraceae emerged as the principal candidate keystone family, forming a large, central module that connected numerous ASVs across hosts. Microbacteriaceae, Nocardioidaceae, and the archaeal family Nitrosopumilaceae also occupied highly connected positions (Faust and Raes 2012). The central role of Nitrosopumilaceae suggests that Archaea, although less diverse in the dataset, may contribute importantly to macroalgal‐associated network connectivity, likely through nitrification and other nitrogen‐cycling processes. Families in such central positions are expected to contribute disproportionately to network stability and connectivity, for example, by mediating nutrient cycling and by engaging in competitive or cooperative interactions that buffer holobionts against environmental fluctuations (Faust and Raes 2012; Banerjee et al. 2018).

Given that modules in microbial co‐occurrence networks can represent distinct ecological niches or life‐history strategies (Ma et al. 2020), the modular structure observed among macroalgal hosts likely reflects the combined influence of host traits and microbial interactions on community assembly. Regarding our third question, the identification of families such as Rubrobacteraceae, Nocardioidaceae, and Nitrosopumilaceae as network candidate keystones indicates that Laminariales holobionts share conserved patterns of functional connectivity and central taxa across the compiled datasets. Future multi‐omics and time‐series studies will be essential to confirm the metabolic roles of these candidate keystone taxa, particularly archaeal lineages such as Nitrosopumilaceae, and to determine how their interactions and functional contributions respond to environmental perturbations (Martiny et al. 2006; Campbell et al. 2019). More broadly, a comprehensive understanding of macroalgal holobionts will require expanding beyond bacterial communities to incorporate microbial eukaryotes, archaea, and viruses, while integrating taxonomic and functional approaches across multiple domains of life (Rousseau et al. 2026). Emerging universal metabarcoding strategies may further facilitate the simultaneous characterization of bacterial, archaeal, and eukaryotic diversity within a single analytical framework (Priest et al. 2025).

## 5 Conclusions

By compiling and re‐analyzing public 16S rRNA datasets using a unified pipeline, this study finds that most research on macroalgal epiphytic bacteria centers on Phaeophyceae (especially Laminariales) and the V4 region of the 16S rRNA gene. This limited scope constrains benchmarking of alternative marker regions and underscores the need for harmonized primer selection, sequencing strategies, and analytical workflows in macroalgal microbiome studies. Standardized methodologies primarily capture ecological patterns associated with the most extensively studied macroalgal hosts.

Our analyses show that host identity, especially at the genus level within Laminariales, consistently structures epiphytic bacterial communities. Laminariales surfaces host distinct assemblages that differ from the surrounding seawater and share a small ASV core across genera, while also displaying genus‐level taxonomic signatures. Functional inference and network analyses showed that Laminariales microbiomes were enriched in aerobic chemoheterotrophy, organic‐matter degradation, and nutrient cycling, with highly connected networks centered on recurrent bacterial and archaeal families such as Rubrobacteraceae, Microbacteriaceae, Nocardioidaceae, and Nitrosopumilaceae. The central, recurring presence of these taxa suggests they are candidate keystone lineages, although experimental validation remains necessary to confirm their ecological roles. These findings indicate that Laminariales‐associated microbiomes are shaped by host filtering and exhibit consistent taxonomic and functional organization. Together, these results create a quantitative framework for future multi‐omics and experimental investigations of macroalgal–microbial associations under environmental change.

## Author Contributions

**J. Z. Florez:** conceptualization, methodology, investigation, visualization, formal analysis, writing – original draft, software, data curation, funding acquisition. **A. Zárate:** investigation, formal analysis, visualization, writing – original draft, methodology, data curation, software. **P. Bruna:** methodology, software, data curation, formal analysis, visualization, writing – original draft. **B. Leyton‐Carcaman:** methodology, software, data curation, formal analysis, visualization, writing – original draft. **V. Molina:** writing – review and editing, writing – original draft. **M. B. Hengst:** writing – review and editing, writing – original draft. **C. Camus:** writing – review and editing, writing – original draft. **M. Abanto:** writing – review and editing, writing – original draft. **L. Barrientos:** writing – review and editing, writing – original draft. **A. H. Buschmann:** writing – original draft, writing – review and editing, conceptualization, funding acquisition.

## Ethics Statement

The authors have nothing to report.

## Conflicts of Interest

The authors declare no conflicts of interest.

## Supporting information


**Figure S1:** Rarefaction curves based on observed ASVs for epiphytic bacterial communities associated with Laminariales. Each line represents an individual sample and depicts the expected ASV richness as a function of sequencing depth (number of reads), estimated by repeated random subsampling. Curves are coloured by macroalgal host genus, enabling comparison of microbial richness accumulation among Laminariales genera across a common gradient of sampling effort.

**Figure S2:** Variance partitioning of epiphytic bacterial community composition. Venn diagram showing the proportion of variance in community structure explained by (A) Host order and oceanic region for the full Phaeophyceae group, and (B) Host genus and oceanic region within the Laminariales order. Percentages indicate the unique and shared fractions for each factor, with the residual corresponding to unexplained variance.

**Figure S3:** Violin plots showing the distribution of the alpha diversity index (i.e., Chao1 richness, Shannon diversity, and inverse Simpson) for epiphytic bacterial communities associated with each macroalgal genus and seawater. Dots represent individual samples, and horizontal bars indicate significant pairwise differences among groups based on Kruskal–Wallis tests followed by the post hoc Tukey HSD test (*p* < 0.001).

**Figure S4:** Sankey diagram showing functional annotations of epiphytic bacterial communities based on FAPROTAX. Links connect functional groups (left) to Sample types (right). The width of each stream is proportional to the relative contribution of ASVs assigned to each functional category within a given host genus and order.

**Figure S5:** Network from seawater ASV presence‐absence data set. Each node represents a macroalgal species. Nodes correspond to individual macroalgal species. For ease of interpretation, identical node colours (fill and border) may recur within the network.

**Figure S6:** Network of microbial co‐occurrence patterns in the bacterial communities, at the family level, associated with the different groups of macroalgae (Phaeophyceae, Chlorophyta and Rhodophyta) and seawater. The nodes (bubbles) represent the ASVs, and edges (lines) represent the strong correlations (Spearman's *p* > 0.75) and statistical significance (FDR‐adjusted *p* < 0.01). Module hubs were coloured by modularity class, and the size of each node is proportional to the BCC value (see Table S4).

**Table S1:** Summary table of the database analysed in this study considering the different macroalgal groups (C‐Chlorophyta, P‐Phaeophyceae, R‐Rhodophyta). Abbreviation: Technology: Sequencing type; Min‐length: ASV determined by minimum length; Max‐length: ASV determined by maximum length; n‐Samples: Total samples; Samples‐rarefaction: Number of rarefaction samples; n‐Features: Total ASVs initially recovered; Features‐rarefaction: Number of ASVs recovered after rarefaction; Max‐depth‐count: Total reads of all ASVs; Deep rarefaction: Number of reads at which the plate is reached in the largest number of samples. Alpha rarefaction: Rarefaction curve evaluation (pass indicates high sample recovery).

**Table S2:** Descriptive summary of the meta‐analysis dataset (*n* = 763 samples). For each variable, the table reports the number of samples in each category (Frequency) and their proportion relative to the total dataset (Percentage).

**Table S3:** Taxonomic composition and network metrics of the co‐occurrence modules identified in Phaeophyceae‐associated bacterial communities. This table complements the genus‐level functional summaries presented in Figure 6.

**Table S4:** Taxonomic composition and network metrics of the co‐occurrence modules identified in bacterial communities associated with different macroalgal groups (Phaeophyceae, Chlorophyta, and Rhodophyta) and seawater. This table complements the family‐level functional summaries presented in Figure S6.

**Table S5:** Species‐level distribution of FAPROTAX‐predicted functional groups associated with bacterial communities inhabiting brown macroalgae. Rows correspond to functional categories inferred using FAPROTAX, and columns represent individual macroalgal species included in the analysis. Values indicate the relative abundance (%) of each predicted function within the bacterial community associated with each host species. This table complements the genus‐level functional summaries presented in Figure 4C.

## Data Availability

The data that support the findings of this study are openly available in public sequence databases, with the individual accession numbers for each study listed in Table S1. The scripts developed for this work were deposited on GitHub (https://github.com/pabruna/epibacterial‐macroalgae‐scripts).
